# Genetic variants in inflammatory cytokines and angiogenesis associated with diabetic nephropathy- A meta-analysis

**DOI:** 10.1186/1471-2164-15-S2-P12

**Published:** 2014-04-02

**Authors:** Khalid Siddiqui, Dhekra Al-Naqeb, Sarah Al-Qassim, Nyla Nazir

**Affiliations:** 1 Diabetes Strategic Research Center, King Saud University, Riyadh, KSA

## Background

Diabetes mellitus (DM) is the most common chronic endocrine disorder, affecting an estimated 371 million people worldwide. It is associated with microvascular and macrovascular complications, including diabetic nephropathy (DN), a primary cause of end-stage renal disease (ESRD). Among the Gulf countries, Saudi Arabia has the highest number of people with diabetes and about 30-40% diabetic patients suffer from DN. Diabetes imposes a large economic burden on healthcare systems. Healthcare expenditures due to diabetes account for 11% of the total medical expenditures in the world in 2011. Mostly, individuals exposed to long durations of diabetes with relatively poor glycemic control develop progressive DN. However, some patients appear to be at increased risk while some will remain relatively protected. Differential disease risk in DN may be partly attributable to genetic susceptibility. A recent review identified genetic variants in the ACE and MTHFR genes to be significantly associated with type 2 diabetes in Arabs [1]. Inflammatory cytokines and angiogenic factors are important modulators in the pathogenesis of DN. We aimed to determine which of the previously investigated genetic variants in these pathways are significantly associated with the development of DN in diabetes and to examine the functional role of these genes.

## Materials and methods

A systematic meta-analysis was undertaken to collectively analyze all studies published till June, 2013 that investigated the association between genetic variants involved in inflammatory cytokines and angiogenesis and the development of DN. Genetic variants associated with DN were selected and analyzed using Comprehensive Meta Analysis software. Pathway analysis of the genes with variants showing significant positive association with DN was performed using Genomatix Genome Analyzer (Genomatix, Munich, Germany).

## Results

After the inclusion and exclusion criteria for this analysis, 34 studies were included in this meta-analysis. 11 genetic variants showed significant positive association with DN in a random-effects meta-analysis. These included genetic variants within or near VEGFA, CCR5, CCL2, IL-1, MMP9, EPO, IL-8, ADIPOQ and IL-10. There are very few studies identifying genetic variants associated with DN in the Arab population. 819C/T genetic variant in IL10 showed protective effect for DN in Tunisian Arabs.

## Conclusions

We are in the process of studying the functional role of the genes showing significant positive association with DN in different pathways like signal transduction and molecular function. The functional relevance of the variants and their pathways can lead to novel biological insights and development of new therapeutic targets.
